# Allometry and Yield Stability of Cereals

**DOI:** 10.3389/fpls.2021.681490

**Published:** 2021-09-10

**Authors:** Jacob Weiner, Yan-Lei Du, Yi-Min Zhao, Feng-Min Li

**Affiliations:** ^1^Department of Plant and Environmental Science, University of Copenhagen, Frederiksberg, Denmark; ^2^State Key Laboratory of Grassland Agroecosystems, Institute of Arid Agroecology, School of Life Sciences, Lanzhou University, Lanzhou, China

**Keywords:** crop growth, allocation, size dependency, appropriate technology, smallholder farmers, breeding strategy

## Abstract

Crop plants grow, and then, they allocate resources to different structures, including seeds and fruits, which represent yield in most crops. We define the yield stability of a genotype as its ability to reduce the effects of temporal variation in resources and conditions on yield production, and we argue that yield stability can be understood in terms of two processes: (1) crop survival and growth (biomass production): the ability of the crop plants to survive and produce biomass under the range of conditions to which it is exposed and (2) the pattern of allocation of this biomass to yield across this range of conditions. Plant breeders and crop physiologists have focused on (1), but much less attention has been paid to (2). We hypothesize that (2) is primarily the result of reproductive allometry: the quantitative relationship between vegetative and reproductive biomass. Ecological theory and the allometric models we present predict a tradeoff between (a) the ability of a genotype to produce yield over a wide variety of conditions and (b) its ability to produce very high yields under optimal or near-optimal conditions. We reanalyze the data from two recent studies, and the results are consistent with this hypothesis. Yield stability in crops corresponds to bet-hedging in evolutionary ecological theory. It is the most appropriate strategy for smallholder farmers in developing countries, a group that comprises most of the farmers in the world. Researchers and crop breeders need to rethink their objectives if they want to develop optimal varieties for these farmers.

## Introduction

One of the most important concepts in agronomy is yield stability, but there is not complete agreement on a definition of this important concept, whether it should be applied to geographical as well as temporal variability, and how to measure it statistically (Eberhart and Russell, 1966; Lin et al., 1986; Cleveland, 2001). A discussion of the different definitions and measures of yield stability is beyond the scope of this short perspective article, but here, we use one of the most widely used and intuitive definitions: the inverse of the variation in yield among years due to year-to-year variation in resources (e.g., water and nutrient availability; Boussakouran et al., 2021) and conditions (e.g., temperature; Tollenaar and Lee, 2002). The evaluation and measurement of yield stability is highly determined by the specific conditions under which it is measured. If conditions are not highly variable, there will be relatively little variation in yield. In addition, there is no reason to assume that a genotype that is stable with respect to variation in one resource or factor will also be relatively stable in response to variation in a different resource or factor. We argue that yield stability can be understood and analyzed in terms of two processes: plant growth and allometric allocation.

Plants grow, and then, they allocate their resources to different functions, among them reproduction, which represents yield for most crops. Yield stability can only be achieved through plant responses in growth and/or reproductive allocation to temporal variation. To produce relatively stable yields, the plant must be able to (1) survive and grow, and then (2) allocate resources to yield formation under all the conditions that it encounters in different seasons. There is a wealth of information about (1), but (2) has been given much less attention, so we focus on this here.

## Allometry

Yield production has been conceptualized and analyzed as a developmental/physiological process (Hay and Porter, 2006). This is certainly a good place to start, but this classical approach does not focus on the quantitative relationship between yield and growth. Allometry is the quantitative relationship between different plant parts, dimensions, or processes, and has been a central analytical framework in biology since it was introduced by J. Huxley in 1932 (Huxley, 1932). In the context of allocation to reproduction, allometry is the quantitative relationship between vegetative (i.e., non-reproductive) and reproductive biomass (Weiner, 2004).

There has been much discussion in the plant ecological literature about how to delimit reproductive biomass. Should it include all structures related to reproduction, e.g., flowering stalks and bracts, even though some of these can photosynthesize, and therefore have aspects of vegetative biomass (Bazzaz and Reekie, 1985)? Because we are primarily addressing cereal crop plants and focusing on yield, we conveniently avoid these potential complications and simply consider reproductive biomass (R) as the mass of grains produced by an individual, i.e., individual yield. We consider all other biomass as vegetative (V). Together, R+V=total plant biomass (T). Relationships among other “yield components” are also of interest, but here, we focus only on these two variables. Ideally, V would include root as well as shoot biomass, but this is not possible in most cases. Researchers have to live with this limit on almost all agricultural studies until effective methods to measure individual root biomass in the field are developed.

Agricultural researchers have used the Harvest Index (R/T) to quantify allocation to yield, and this corresponds to what ecologists have called Reproductive Effort (Bazzaz and Reekie, 1985). But the Harvest Index is allometric; i.e., it changes with size, and therefore, it could be misleading to treat HI as a trait for breeding. Rather, an allometric approach to allocation is more appropriate (Qin et al., 2013). Allometric allocation is the most likely explanation for variation in Harvest Index from year to year (e.g., Singh and Stoskopf, 1971) or in response to changes in crop density (Qin et al., 2013; Li et al., 2015). If biomass accumulation varies from season to season, then it is the allometric program, not the Harvest Index as such, which is being selected in nature or by plant breeders.

### The Allometry of Yield

Evidence is accumulating in support of the hypothesis that the allometric relationship between individual plant size (V) and potential yield (R) is genetically fixed (Weiner et al., 2009). There is extensive plasticity in growth rate and developmental timing (e.g., initiation of flowering), but, at any given size, a plant has a fixed potential for seed production. The plant does not always achieve this potential reproductive output because external factors, such as frost, pests, or disease can intervene and prevent this. This allometric approach is especially relevant to annual crops, which generally follow the optimal reproductive allocation strategy first described by Cohen (1968): Plants allocate all resources to growth early in the season, and then, at some point, they switch to investing all resources into reproduction (yield formation).

**Figure 1:**
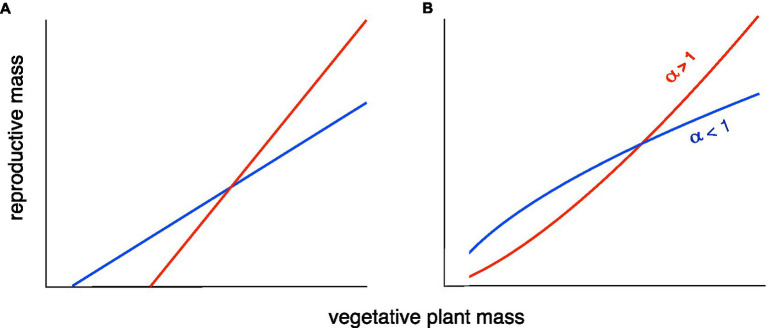
Two versions of two types of allometric relationship between plant reproductive output (yield) and size [after Weiner et al. (2009) and Bonser (2020)]. **(A)** A linear relationship with a positive X-intercept (minimum size for reproducing). **(B)** A classical allometric relationship, Y=βX^α^. In both cases, there is a tradeoff between the maximum reproduction at large size and reproduction at smaller sizes. The red lines result of selection for maximal performance under good conditions, as in the breeding of high-yielding varieties for high-input agriculture. The blue lines result from a “bet-hedging” (risk reduction) strategy to reduce the probability of a very bad outcome. The latter strategy will be advantageous when there is large temporal and/or spatial variation in conditions, including some very unfavorable conditions, and is the most appropriate strategy for smallholder farmers in developing countries. The tradeoff can be visualized as a constraint on the area under the curve.

A review of R–V relationships in plants (Weiner et al., 2009) found two common patterns: A linear relationship, usually with a positive X-intercept, representing the minimum size for reproduction (Figure 1A), or a classical allometric relationship of the form Y=βX^α^, usually plotted and analyzed as log Y=log β+α log X to make it linear, but shown here on a linear scale (Figure 1B).

**Figure 2:**
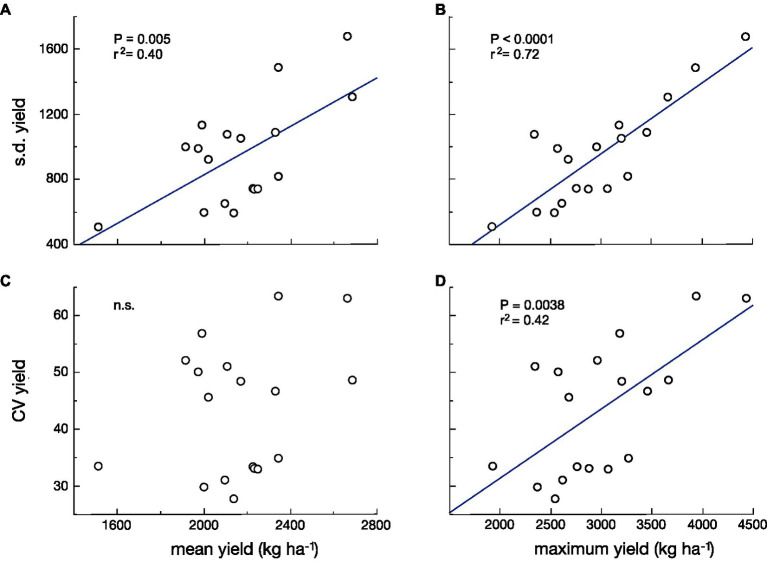
The standard deviation (s.d.; **A,B**) and the coefficient of variation (CV; **C,D**) of yield *vs*. mean yield **(A,C)** and *vs*. maximum yield (B,D) for 18 cultivars of wheat grown in three environments. The relationships with the highest yield are stronger and more significant than those with mean yield [Data from Du et al. (2020)].

In both models, there is less change in reproductive output per unit change in size under the bet-hedging strategy than the maximal yield strategy across the range of plant sizes. We have recently argued that there is a tradeoff between yield and yield stability (Du et al., 2020). Here, we revise this and argue that the tradeoff is not necessarily between mean yield and yield stability, but between maximum yield and yield stability. This hypothesis is supported by a reanalysis of the data in our previous paper. The fit between the standard deviation (s.d.) of yield and maximum yield of 18 cultivars grown in three different environments is much stronger than that between s.d. of yield and mean yield (Figures 2A,B). It can be argued that the s.d. can be expected to increase with the mean, so many analyses of yield stability use the coefficient of variation (CV=s.d./mean; Lin et al., 1986) in yield as the measure of yield stability. If we do this, the argument becomes even stronger. There is no significant relationship between CV of yield and mean yield, but there is a highly significant relationship between CV of yield and maximum yield (Figures 2C,D).

**Figure 3:**
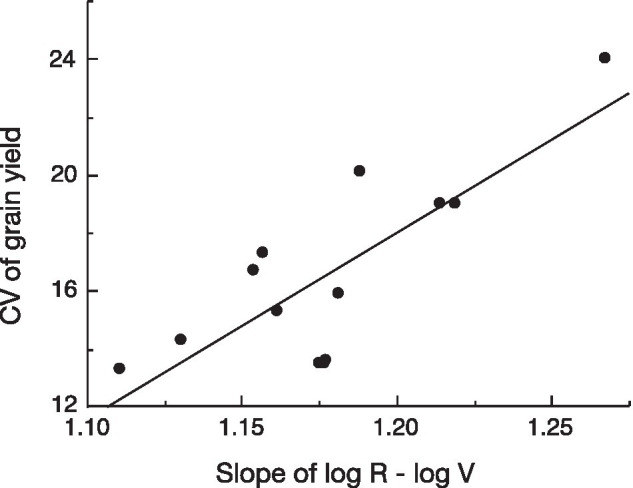
Linear regression of coefficient of variation (CV) of yield *versus* slope of the log (reproductive biomass, R)–log (vegetative biomass, V) relationship for 13 genotypes of winter wheat grown at nine environments in Gansu Province, China [data from Zhao (2020); Yan-Lei Du, unpublished]; *p*=0.0012.

To test the assumption behind our hypothesis that variation in yield reflects the slope of the log R–log V relationship when this model fits the observations, we analyzed the recent data on variation in yield *vs*. the slope of the log R–log V of 13 genotypes of winter wheat grown in nine environments in Gansu Province, China (Zhao, 2020; Yan-Lei Du, unpublished). There was a significant positive relationship between variation in yield and the allometric slope (exponent; Figure 3).

## Discussion

Our models and results represent a first step in clarifying the relationship between yield and yield stability allometrically. The data presented here are preliminary, and further test of our hypothesis with larger data sets covering a wide range of conditions is much needed. It is worth noting that in our theoretical examples above (Figure 1), the average Harvest Index for both genotypes could be similar, showing that the Harvest Index is not the optimal way to interpret changes in allocation to yield.

It makes sense that maximum yield is a better parameter than mean yield for some modeling purposes, because whether or not data show evidence of a tradeoff between mean yield and yield stability is highly dependent on the distribution of conditions (and therefore of plant sizes) over different seasons. The maximum yield under good conditions (also called yield potential) is more directly a function of the genotype and less a function of the specific conditions (seasons or locations) used to determine it, so it serves as a better parameter for modeling than does mean yield. This is likely to be the case even if the highest-yielding conditions in a specific study are not ideal, because the highest-yielding conditions will be the closest to optimal. If all the conditions under which yield stability is determined are very poor, however, this will not be the case and our argument will not hold.

The fact that any measure of yield stability is highly dependent on the variation in conditions used to estimate it could explain why some studies show a positive relationship between yield potential and stability. If the variation in conditions under which yield stability is evaluated is relatively small and the conditions are generally favorable, as is often the case in high-input agricultural systems, this is a possible result. If, on the other hand, the variation in conditions is great and includes some very unfavorable conditions, as in low-input farming systems, such as small holder farming systems in developing countries, the tradeoff between maximum yield and yield stability will appear according to our models.

Both models of the R–V relationship shown in Figure 1 have been documented in a review of R–V relationships in plants (Weiner et al., 2009). For wild species that show the classical allometric model (Figure 1B), the allometric slope (exponent) was always ≤1. We hypothesize that breeding for high yields in high-input agricultural systems has produced cultivars that have an allometric slope >1, increasing maximum yield under good conditions at the expense of yield under poorer conditions. In economics, such a pattern would be called an “economy of scale” (Stigler, 1958). A slope of <1 means that smaller individuals have a higher Reproductive Effort or “reproductive economy” (Aarssen, 2015).

### Clarifying the Research Objectives

There has been much criticism of well-meaning attempts by agricultural researchers from developed countries to improve agricultural production in developing countries by applying knowledge obtained back home under high-input conditions in a totally different socio-economic context. It is not just a question of the resources and technology available: These differences are associated with different societal and agricultural needs and priorities. We argue that the focus on maximizing yield itself is an example of the misapplication of agronomic research from the (mostly temperate and relatively wealthy) developed societies to the (mostly tropical and poor) developing counties. “Appropriate technology” means appropriate for the needs of the farmers and consumers, so the objective of research, not only the methods, must be appropriate. Most of the world’s farmers are small holders in developing countries. They do not need the ability to produce very high yields under very high resource input and good conditions as much as they need the ability to produce reasonable yields despite their limited resources and the vagaries of weather, pest attacks, etc., to which their crops are exposed. Our results provide evidence that there is a tradeoff between these two objectives, and we urge agricultural researchers and breeders working in developing countries to focus on the latter if they are to find strategies and solutions for smallholder farmers in the developing world. These farmers do not present a lucrative market for private breeding companies, so commercial breeding does not serve their needs. Alternative models for funding research and pursuing plant breeding to serve smallholder are needed if research is to contribute to lifting farmers out of poverty and providing foodstuffs for their regions.

## Data Availability Statement

The original contributions presented in the study are included in the article/supplementary material, further inquiries can be directed to the corresponding author.

## Author Contributions

JW led the writing of the paper. Y-LD, F-ML, and JW developed the ideas. Y-MZ provided the data. All authors contributed to the article and approved the submitted version.

## Funding

This research was supported by the National Nature Science Foundation of China (31670401), Science and Technology Planning Project of Gansu Province (18JR3RA281), “111” Project (BP0719040), and the Fundamental Research Funds for the Central Universities (lzujbky-2021-sp42).

## Conflict of Interest

The authors declare that the research was conducted in the absence of any commercial or financial relationships that could be construed as a potential conflict of interest.

## Publisher’s Note

All claims expressed in this article are solely those of the authors and do not necessarily represent those of their affiliated organizations, or those of the publisher, the editors and the reviewers. Any product that may be evaluated in this article, or claim that may be made by its manufacturer, is not guaranteed or endorsed by the publisher.
